# Presentation and Outcomes of Adults With Overdose-Related Out-of-Hospital Cardiac Arrest

**DOI:** 10.1001/jamanetworkopen.2023.41921

**Published:** 2023-11-07

**Authors:** Vidhushei Yogeswaran, Christopher Drucker, Kosuke Kume, Amy Poel, Nicole Yarid, Sarah Leyde, Thomas D. Rea, Neal A. Chatterjee

**Affiliations:** 1 Department of Medicine, University of Washington, Seattle, Washington; 2 Public Health–Seattle & King County Division of Emergency Medical Services, Seattle, Washington; 3 King County Medical Examiner’s Office, Seattle, Washington

## Abstract

**Importance:**

Drug overdose (OD) is a public health challenge and an important cause of out-of-hospital cardiac arrest (OHCA). Existing studies evaluating OD-related OHCA (OD-OHCA) either aggregate all drugs or focus on opioids. The epidemiology, presentation, and outcomes of drug-specific OHCA are largely unknown.

**Objective:**

To evaluate the temporal pattern, clinical presentation, care, and outcomes of adult patients with OHCA overall and according to the drug-specific profile.

**Design, Setting, and Participants:**

This cohort study of adults with OHCA in King County Washington was conducted between January 1, 2015, and December 31, 2021. Etiology of OHCA was determined using emergency medical service, hospital, and medical examiner records. Etiology was classified as non-OD OHCA or OD-OHCA, with drug-specific profiles categorized as (1) opioid without stimulant, (2) stimulant without opioid, (3) opioid and stimulant, or (4) all other nonstimulant, nonopioid drugs. Statistical analysis was performed on July 1, 2023.

**Exposure:**

Out-of-hospital cardiac arrest.

**Main Outcomes and Measures:**

The primary outcome was survival to hospital discharge. The secondary outcome was survival with favorable functional status defined by Cerebral Performance Category 1 or 2 based on review of the hospital record.

**Results:**

In this cohort study, there were 6790 adult patients with emergency medical services–treated OHCA from a US metropolitan system. During the 7-year study period, there were 702 patients with OD-OHCA (median age, 41 years [IQR, 29-53 years]; 64% male [n = 450] and 36% female [n = 252]) and 6088 patients with non-OD OHCA (median age, 66 years [IQR, 56-77 years]; 65% male [n = 3944] and 35% female [n = 2144]). The incidence of OD-OHCA increased from 5.2 (95% CI, 3.8-6.6) per 100 000 person-years in 2015 to 13.0 (95% CI, 10.9-15.1) per 100 000 person-years in 2021 (*P* < .001 for trend), whereas there was no significant temporal change in the incidence of non-OD OHCA (*P* = .30). OD-OHCA were more likely to be unwitnessed (66% [460 of 702] vs 41% [2515 of 6088]) and less likely to be shockable (8% [56 of 702] vs 25% [1529 of 6088]) compared with non-OD OHCA. Unadjusted survival was not different (20% [138 of 702] for OD vs 18% [1095 of 6088] for non-OD). When stratified by drug profile, combined opioid-stimulant OHCA demonstrated the greatest relative increase in incidence. Presentation and outcomes differed by drug profile. Patients with stimulant-only OHCA were more likely to have a shockable rhythm (24% [31 of 129]) compared with patients with opioid-only OHCA (4% [11 of 295]) or patients with combined stimulant-opioid OHCA 5% [10 of 205]), and they were more likely to have a witnessed arrest (50% [64 of 129]) compared with patients with OHCA due to other drugs (19% [14 of 73]) or patients with combined stimulant-opioid OHCA (23% [48 of 205]). Patients with a combined opioid-stimulant OHCA had the lowest survival to hospital discharge (10% [21 of 205]) compared with patients with stimulant-only OHCA (22% [29 of 129]) or patients with OHCA due to other drugs (26% [19 of 73]), a difference that persisted after multivariable adjustment.

**Conclusions and Relevance:**

In a population-based cohort study, the incidence of OD-OHCA increased significantly from 2015 to 2021, with the greatest increase observed among patients with a combined stimulant-opioid OHCA. Presentation and outcome differed according to the drug-specific profile. The combination of increasing incidence and lower survival among among patients with a opioid-stimulant OHCA supports prevention and treatment initiatives that consider the drug-specific profile.

## Key Points

**Question:**

Are there differences in the temporal pattern, presentation, or outcomes for drug-specific etiology of out-of-hospital cardiac arrest (OHCA) due to overdose (OD)?

**Findings:**

In a population-based cohort study of 6790 adult patients with emergency medical services–treated OHCA from a US metropolitan system, the incidence of OD-OHCA more than doubled from January 1, 2015, to December 31, 2021, compared with the incidence of non-OD OHCA (ie, no significant change); OHCA due to combined opioid-stimulant etiology experienced the lowest survival among drug-specific profiles.

**Meaning:**

The combination of increasing incidence and lower survival among OHCA secondary to combined opioid-stimulant substance use frames an urgent need for prevention and resuscitation strategies in this population.

## Teaser

This cohort study evaluates survival and outcomes of adult patients with drug overdose–related out-of-hospital cardiac arrest (OHCA) compared with patients with OHCA unrelated to overdose.

## Introduction

Substance use disorder is a major public health crisis responsible for more than 100 000 deaths each year in the United States.^1^ Substances such as opioids, stimulants, and benzodiazepines can be acutely lethal and are an important cause of overdose-related out-of-hospital cardiac arrest (OD-OHCA).^2^ Substance use has been associated with a range of cardiopulmonary pathophysiological conditions, including arrhythmogenesis, vascular dysfunction, atherosclerosis, respiratory depression, and out-of-hospital cardiac arrest (OHCA).^2^ While opioid-associated OHCA has historically comprised the majority of cases of OD-OHCA,^2^ patterns of drug use are changing, with an increasing prevalence of synthetic opioids and stimulants underscoring increasing mortality among individuals with substance use disorder.^3,4^

Previous studies evaluating OD-OHCA^5,6,7,8,9^ have frequently grouped substances together without distinguishing drug-specific profiles, have relied on nonsystematic toxicology screens or inferential evidence for OD to determine OHCA etiology, and have focused on opioid-related OHCA. Currently, to our knowledge, little is known about the drug-specific characteristics of OD-OHCA in the contemporary era of the substance use epidemic. Such knowledge could have implications for acute resuscitation treatment, tailored postresuscitation care, and public health efforts to reduce morbidity and mortality related to substance use disorder. In this study, we used a longitudinal, population-based OHCA registry, in conjunction with a medical examiner evaluation, to evaluate the incidence, presentation, resuscitation care, and clinical outcomes of adult patients with OD-OHCA in the context of all types of OHCA.

## Methods

### Study Design, Population, and Setting

This investigation was a retrospective cohort study of persons 18 years or older who experienced nontraumatic OHCA and received attempted resuscitation by emergency medical services (EMS) between January 1, 2015, and December 31, 2021, in greater King County, Washington, excluding Seattle. The study was approved by the institutional review boards of the University of Washington and Public Health–Seattle & King County and used the Strengthening the Reporting of Observational Studies in Epidemiology (STROBE) reporting guideline for observational research.^10^ Informed consent was waived because the data were deidentified.

Greater King County is a metropolitan region with a population of 1.5 million persons residing in urban, suburban, and rural areas. Individuals can activate the EMS response by calling 9-1-1 and contacting a telecommunicator, who then uses questions about consciousness and breathing to identify suspected OHCA. For those with suspected OHCA, the telecommunicator coaches the layperson using an automated external defibrillator and performing cardiopulmonary resuscitation (CPR). Law enforcement responds to the suspected OHCA in some, though not all, communities in King County. The EMS response is a 2-tiered system. The first tier comprises firefighter emergency medical technicians trained in CPR and automated external defibrillator use. The second tier comprises critical care paramedics who are dispatched for more serious illness, including suspected OHCA. The scope of paramedic practice includes electrocardiogram rhythm interpretation, manual defibrillation, intravenous and intraosseous drug administration, and advanced airway management. Patients who achieve a return of spontaneous circulation are then transported to 1 of 12 hospitals, each equipped with intensive care unit and coronary catheterization facilities.

### Data Sources and Definitions

The EMS system maintains a registry of EMS-treated cardiac arrests. Information regarding arrest rhythm, arrest etiology, presentation, treatment, and outcome is collected from dispatch audio recordings, defibrillator electronic data, prehospital and hospital records, death certificates, and Medical Examiner’s Office (MEO) reports. The etiology of the OHCA (ie, OD, primary cardiac, other medical, and traumatic) relied on clinical review of all data sources, including MEO reports and toxicology.

OD classification of OHCA was based on systematic toxicology and autopsy data for decedents and systematic review of EMS reports, clinical history, and urine toxicology for survivors. For decedents, the King County MEO investigates all sudden, unexpected, violent, suspicious, or unnatural deaths, including suspected drug overdoses.^11^ These postmortem examinations include analysis of the death scene, autopsy, and toxicology evaluation to determine the cause of death.^11^ The MEO collects blood and vitreous samples from these cases, except in situations of advanced postmortem decomposition.^12^ The Washington State Patrol Toxicology Laboratory then tests these postmortem blood and vitreous samples using head-space gas chromatography, screening for classes of drugs by enzyme-multiplied immunoassay technique or gas chromatography–mass spectrometry and confirming and quantifying the specific drug by gas chromatography–mass spectrometry and liquid chromatography–mass spectrometry.^12^ Novel fentanyl analogs and other less common synthetic drugs cannot be detected by these methods, and blood samples specimens are then submitted to a private laboratory (NMS Labs, Inc) for further evaluation.^12^ The specific drugs tested for include opioids, stimulants, and synthetic drugs.^12,13^ Drug-specific profiles and quantification are then listed on the final MEO toxicology report.

For survivors of OHCA, adjudication of OD etiology was performed using systematic adjudication of multiple data sources, including EMS reports (including information regarding drug paraphernalia and bystander reports of drug use), hospital care records, and urine toxicology, when available. For survivors, an OD etiology was considered when there was urine toxicology supportive of etiology or, in a minority of cases (n = 2), data implicating illicit drug use in hospital records or EMS reports in the absence of an alternative etiology. Taken together, there was consensus regarding OD as the primary etiology in OHCA in 96% of cases (682 of 713). In the remaining cases in which etiology was indeterminate (n = 31), a 3-physician review (V.Y., N.A.C., and T.D.R.) was performed to adjudicate etiology. In this circumstance, 20 cases were adjudicated by consensus as drug-related OHCA, and 12 cases were classified as non-OD OHCA.

Each drug available from the MEO or hospital toxicology (for full list, see the eMethods in Supplement 1) was abstracted according to the US Drug Enforcement Administration 2020 Drugs of Abuse Resource Guide.^14^ The OD profile was classified into 4 categories based on the prevalent epidemiology of substance use: (1) opioid without stimulant, (2) stimulant without opioid, (3) combined opioid and stimulant, and (4) all other nonstimulant, nonopioid drugs. Opioids included fentanyl (including novel fentanyl analogs), heroin, hydromorphone, methadone, opium, codeine, and oxycodone. Stimulants included amphetamines, methamphetamines, and cocaine.

### Outcome Measures

The primary outcome was survival to hospital discharge. The secondary outcome was survival with favorable functional status defined by Cerebral Performance Category 1 or 2 based on review of the hospital record.

### Statistical Analysis

Statistical analysis was performed on July 1, 2023. Descriptive characteristics were calculated as numbers and total percentage of their category. We estimated crude and age-adjusted incidence rates and 95% CIs by calculating the number of patients with OHCA per 100 000 adult population per year in the study community according to drug status. Age adjustment was performed by direct standardization to the US 2000 standard population with age-adjustment groups of 18 to 24, 25 to 44, 45 to 64, and 65 years of age or older.^15^ We used joinpoint regression analysis to test for trend in the change of annual incidence.^16^

We undertook 2 sets of comparisons. The first compared characteristics and outcomes of aggregated cases of OD-OHCA vs cases of non-OD OHCA. The second evaluated characteristics and outcomes for cases of drug-specific OHCA, comparing the 4 exclusive drug groups.

The association of OD etiology with survival to hospital discharge was evaluated using logistic regression models. We first report the results of unadjusted models, followed by models adjusted for age and sex, to understand how these basic demographic characteristics confound OD outcome associaton. In subsequent multivariable models (Utstein adjusted), we added location (public or private), witness status, bystander CPR, public access defibrillator application, and initial rhythm because these covariates are known to be associated with survival after OHCA in accordance with the Utstein framework.^17^ In models evaluating drug-specific OHCA, opioid-related OHCA was used as a reference because it was the largest group. Statistical analysis was conducted using SPSS, version 27. A 2-tailed *P* < .05 was considered to indicate statistical significance.

## Results

### Incidence of OHCA and Proportion of Cases of OHCA With OD Etiology

During the 7-year study period, there were 6790 adult patients of EMS-treated OHCA consisting of 702 patients with OD-OHCA (median age, 41 years [IQR, 29-53 years]; 36% female [n = 252]) and 6088 non-OD OHCA (median age, 66 years [IQR, 56-77 years]; 35% female [n = 2144]). Among OHCA deaths, toxicology testing during MEO investigation was the primary means to determine OD etiology (eTable 1 in Supplement 1). There was significant correlation between MEO OD etiology classification and hospital record review among those who survived to hospital admission but then died during hospitalization (κ coefficient = 0.84). The age-standardized incidence of OD-OHCA increased significantly over the study period from 5.2 (95% CI, 3.8-6.6) per 100 000 person-years in 2015 to 13.0 (95% CI, 10.9-15.1) per 100 000 person-years in 2021 (*P* < .001 for trend) (Figure; eTable 2 in Supplement 1). By comparison, there was no significant change in the incidence of non-OD OHCA over the study period (69.1 [95% CI, 64.2-74.0] per 100 000 person-years in 2015 and 77.2 [95% CI, 72.4-82.1] per 100 000 person-years in 2021; *P* = .30 for trend). After further stratification of OD-OHCA by drug-specific profile, there was a temporal increase in the incidence of opioid-only, stimulant-only, and the combination of opioid and stimulant OHCA, with the greatest relative increase noted for OHCA secondary to combined opioid and stimulant use (Figure). There was no temporal change in OD-OHCA due to nonopioid, nonstimulant etiology.

**Figure.**
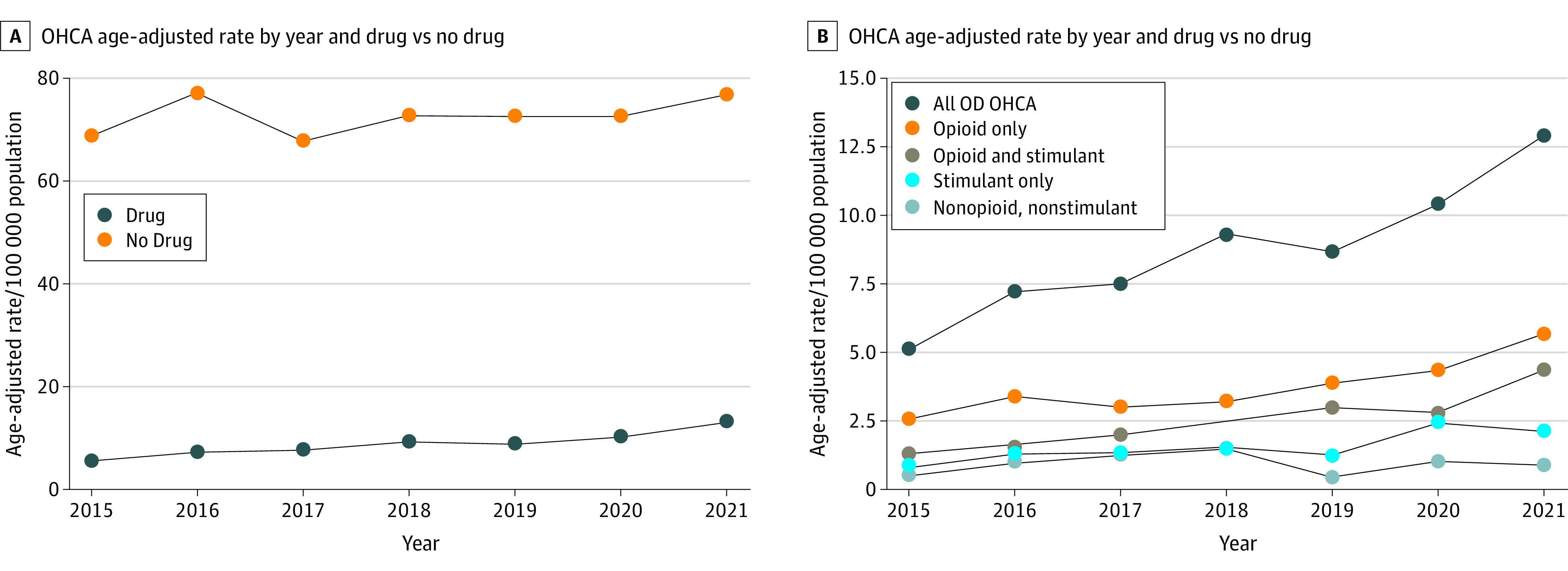
Incidence Rate of Out-of-Hospital Cardiac Arrest (OHCA) in King County Between 2015 and 2021, Stratified by Overdose (OD) Drug Status and Drug-Specific Profiles

### Characteristics and Outcomes of Patients With OHCA According to Drug Overdose Status

Table 1 summarizes the demographics, circumstances, care, and outcomes stratified by OD status and drug-specific OD profile. Compared with non-OD OHCA, individuals with OD-OHCA were younger (median age, 41 years [IQR, 29-53 years] vs 66 years [IQR, 56-77 years]); OD-OHCA was more likely to occur at home (80% [560 of 702] vs 69% [4176 of 6088]) and not be witnessed (66% [460 of 702] vs 41% [2515 of 6088]). The initial presenting arrest rhythm was less likely to be shockable for patients with OD-OHCA compared with patients with non–OD-OHCA (8% [56 of 702] vs 25% [1529 of 6088]). There were similar proportions of bystander CPR and automated external defibrillator application prior to EMS arrival. EMS response times were similar between OD-OHCA and non-OD OHCA. Forty percent of patients with OD-OHCA (278 of 702) and 40% of patients with non-OD OHCA (2460 of 6088) were admitted to the hospital. Among those admitted to the hospital, the proportion receiving targeted temperature management was 55% (ie, 1343 of 2460 patients with non-OD OHCA and 154 of 278 patients with OD-OHCA) regardless of OD status, and a smaller proportion of patients with OD-OHCA received coronary angiography (2% [16 of 278] vs 38% [923 of 2460]).

**Table 1.** OHCA Presentation, Resuscitation Care, and Outcomes, Stratified by Overdose Etiology and Drug-Specific Profiles

Characteristic	Non-OD OHCA (n = 6088)	OD-OHCA (n = 702)	OD drug-specific profile (n = 702)			
			Opioid only (n = 295)	Stimulant only (n = 129)	Combined opioid and stimulant (n = 205)	Other drug (n = 73)
Baseline						
Age (IQR), y	66 (56-77)	41 (29-53)	37 (29-53)	47 (36-54)	37 (29-49)	47 (33-54)
Sex, No. (%)						
Female	2144 (35)	252 (36)	105 (36)	35 (27)	74 (36)	38 (52)
Male	3944 (65)	450 (64)	190 (64)	94 (73)	131 (64)	35 (48)
Initial presentation						
Location, No. (%)						
Public, indoors	414 (7)	36 (5)	13 (4)	7 (5)	11 (5)	5 (7)
Public, outdoors	558 (9)	93 (13)	27 (9)	25 (19)	35 (17)	6 (8)
Home	4176 (69)	560 (80)	252 (85)	95 (74)	155 (76)	58 (80)
Long-term care facility	940 (15)	13 (2)	3 (1)	2 (2)	4 (2)	4 (5)
Bystander witnessed, No. (%)	2821 (46)	191 (27)	65 (22)	64 (50)	48 (23)	14 (19)
EMS witnessed	752 (12)	51 (7)	9 (3)	10 (8)	15 (7)	17 (21)
Unwitnessed	2515 (41)	460 (66)	221 (75)	55 (43)	142 (69)	42 (59)
Bystander CPR^a^	4155 (78)	554 (85)	260 (91)	93 (78)	161 (85)	40 (71)
AED applied	702 (12)	92 (13)	34 (12)	19 (15)	33 (15)	6 (8)
Shockable rhythm	1529 (25)	56 (8)	11 (4)	31 (24)	10 (5)	4 (5)
EMS care, No. (%)						
BLS response interval, median, (IQR), s^b^	303 (239-379)	302 (240-380)	304 (247-376)	296 (218-386)	292 (239-355)	325 (269-437)
ALS response interval, median (IQR), s^c^	486 (374-627)	490 (366-622)	490 (363-628)	484 (372-597)	487 (366-621)	502 (362-688)
Epinephrine	4870 (80)	506 (72)	194 (66)	100 (78)	160 (78)	52 (70)
Naloxone^d^	53 (1)	135 (19)	82 (28)	10 (8)	33 (16)	10 (14)
Advanced airway	5337 (88)	568 (81)	215 (73)	116 (90)	175 (85)	62 (85)
Hospital care, No. (%)						
Admitted to hospital	2460 (40)	278 (40)	116 (39)	64 (50)	63 (31)	35 (48)
TTM use^e^	1343 (55)	154 (55)	62 (53)	37 (58)	41 (65)	14 (40)
Coronary angiography^e^	923 (38)	16 (6)	4 (3)	10 (16)	2 (3)	0
Outcomes, No. (%)						
Survived to hospital discharge	1095 (18)	138 (20)	69 (23)	29 (22)	21 (10)	19 (26)
Favorable neurologic outcome (CPC 1 or 2)	1013 (17)	127 (18)	66 (22)	25 (19)	19 (9)	17 (24)

Abbreviations: AED, automatic external defibrillator; ALS, advanced life support; BLS, basic life support; CPC, Cerebral Performance Category; CPR, cardiopulmonary resuscitation; EMS, emergency medical services; OD, overdose; OD-OHCA, overdose-related out-of-hospital cardiac arrest; TTM, targeted temperature management.

^a^ Bystander CPR information was available for 5336 patients (88%) in the no-drug category and for 651 patients (93%) in the drug-related category, with available data on 286 patients (97%) in the opioid-only category, 119 patients (92%) in the stimulant-only category, 190 patients (93%) in the combined stimulant and opioid category, and 56 patients (78%) in the other-drug category.

^b^ Information on BLS response interval was available for 99% of patients (6030 in the no-drug category and 697 in the drug-related category) with information available on 99% of patients (n = 293) in the opioid-only category, 98% of patients (n = 126) in the stimulant-only category, 100% of patients (n = 205) in the combined stimulant and opioid category, and 100% of patients (n = 73) in the other-drug category.

^c^ Information on ALS response interval was available for 97% of patients (5914 in the no-drug category and 682 in the drug-related category) with information on 97% of patients (n = 286) in the opioid-only category, 96% of patients (n = 124) in the stimulant-only category, 98% of patients (n = 201) in the combined stimulant and opioid category, and 97% of patients (n = 71) in the other-drug category.

^d^ When administered, naloxone was administered after return of pulse by EMS.

^e^ Proportions are reported as a percentage of those admitted to the hospital for a given category.

Survival to hospital discharge did not differ by OD status (18% [1095 of 6088] ] vs 20% [138 of 702]; Table 1) (unadjusted odds ratio [OR], 1.12 [95% CI, 0.92-1.37] for OD-OHCA compared with non-OD OHCA; Table 2). Adjustment for Utstein covariates markedly affected the association of OD status with clinical outcomes, given the substantial differences in demographics (ie, age) and circumstances (ie, witnessed status and presenting rhythm) between OD-OHCA and non-OD OHCA. Compared with non-OD OHCA, the OR of survival to hospital discharge for OD-OHCA was 0.67 (95% CI, 0.54-0.83) after adjustment for age and sex, and yet the OR was 1.59 (95% CI, 1.23-2.04) with additional multivariable adjustment for Utstein covariates. Similar results were observed for the association of OD status and survival to hospital discharge with favorable neurological outcome (Table 2).

**Table 2.** Association of Overdose Status With Clinical Outcomes After OHCA

Model^a^	Survival to hospital discharge			Favorable neurological outcome		
	Yes	No	OR (95% CI)	Yes	No	OR (95% CI)
Unadjusted						
Non-OD OHCA	1095	4993	1 [Reference]	1013	5075	1 [Reference]
OD-OHCA	138	564	1.12 (0.92-1.37)	127	575	1.11 (0.90-1.36)
Age and sex adjusted						
Non-OD OHCA	NA	NA	1 [Reference]	NA	NA	1 [Reference]
OD-OHCA	NA	NA	0.67 (0.54-0.83)	NA	NA	0.66 (0.53-0.83)
Utstein adjusted						
Non-OD OHCA	NA	NA	1 [Reference]	NA	NA	1 [Reference]
OD-OHCA	NA	NA	1.59 (1.23-2.04)	NA	NA	1.65 (1.26-2.14)

Abbreviations: NA, not applicable; OD, overdose; OD-OHCA, overdose-related out-of-hospital cardiac arrest; OR, odds ratio.

^a^ Utstein adjusted models were adjusted for age, sex, public location, witness status, bystander cardiopulmonary resuscitation, automatic external defibrillator application, and initial rhythm.

### Characteristics and Outcomes of Patients With OHCA According to Drug-Specific Overdose Profile

Differences in demographic characteristics across drug-specific categories were not observed (Table 1). Patients with stimulant-only OHCA were more likely to have a bystander witnessed arrest (50% [64 of 129]) compared with patients with OHCA due to other drugs (19% [14 of 73]) or patients with combined stimulant-opioid OHCA (23% [48 of 205]) and were more likely to present with a shockable initial rhythm (24% [31 of 129]) compared with patients with opioid-only OHCA (4% [11 of 295]) or patients with combined stimulant-opioid OHCA 5% [10 of 205]). With regard to hospital care, targeted temperature management was deployed variably across groups (40% [14 of 35] to 65% [41 of 63]) with a similar gradient of coronary angiography use (from 0% of patients with OHCA due to other drugs [0 of 73] to 16% of patients with stimulant-only OHCA [10 of 129]).

Differences in outcome according to drug-specific etiology were observed. At the end of EMS care, patients with combined opioid and stimulant OHCA were least likely to be alive compared with other drug-specific OHCA (31% [63 of 205] vs 39% [116 of 295] vs 50% [64 of 129] for other OD profiles). Likewise, patients with OHCA with combined opioid and stimulant use had the lowest survival to hospital discharge among the OD profiles (10% [21 of 205]) compared with patients with stimulant-only OHCA (22% [29 of 129]) or patients with OHCA due to other drugs (26% [19 of 73]) (Table 1) (unadjusted OR, 0.37 [95% CI, 0.22-0.62]; Table 3). In age- and sex-adjusted models, the survival odds of the combined opioid-stimulant group with respect to the opioid-only group was unchanged (OR, 0.37 [95% CI, 0.22-0.62]); neither the stimulant-only group nor the other-drug-related group showed a significant difference in OHCA survival when compared with the opioid group (Table 3). In further multivariable models that included adjustment for presenting rhythm and witness status, the combined opioid and stimulant group still had the lowest survival odds (OR, 0.26 [95% CI, 0.15-0.47]), while the stimulant-only group had a lower odds of survival to hospital discharge (OR, 0.49 [95% CI, 0.27-0.89]) compared with the opioid-only group. Results were similar for the outcome of survival with favorable neurological outcome (Table 3).

**Table 3.** Association of Drug-Specific Profiles With Clinical Outcomes After Out-of-Hospital Cardiac Arrest

Model^a^	Survival to hospital discharge			Favorable neurological outcome		
	Yes	No	OR (95% CI)	Yes	No	OR (95% CI)
Unadjusted						
Opioid (reference)	69	226	1 [Reference]	66	229	1 [Reference]
Stimulant	29	100	0.93 (0.57-1.52)	25	104	0.83 (0.50-1.40)
Opioid + stimulant	21	184	0.37 (0.22-0.62)	19	186	0.35 (0.21-0.61)
Other	19	54	1.13 (0.63-2.04)	17	56	1.05 (0.57-1.93)
Age and sex adjusted						
Opioid (reference)	NA	NA	1 [Reference]	NA	NA	1 [Reference]
Stimulant	NA	NA	0.96 (0.58-1.58)	NA	NA	0.85 (0.51-1.44)
Opioid + stimulant	NA	NA	0.37 (0.22-0.62)	NA	NA	0.35 (0.20-0.61)
Other	NA	NA	1.10 (0.61-1.98)	NA	NA	0.98 (0.53-1.81)
Utstein adjusted						
Opioid (reference)	NA	NA	1 [Reference]	NA	NA	1 [Reference]
Stimulant	NA	NA	0.49 (0.27-0.89)	NA	NA	0.42 (0.23-0.79)
Opioid + stimulant	NA	NA	0.26 (0.15-0.47)	NA	NA	0.26 (0.14-0.48)
Other	NA	NA	0.74 (0.37-1.47)	NA	NA	0.69 (0.34-1.40)

Abbreviations: NA, not applicable; OR, odds ratio.

^a^ Utstein-adjusted models were adjusted for age, sex, public location, witness status, bystander cardiopulmonary resuscitation, automatic external defibrillator application, and initial rhythm.

## Discussion

In this contemporary population-based cohort of nearly 7000 adult patients with OHCA with systematic adjudication of OD profiles, our study provides insight into drug-specific presentation, resuscitation care, and clinical outcomes. The incidence of OD-OHCA more than doubled from 2015 to 2021 with an almost 4-fold increase in OHCA associated with the combination of opioids and stimulants. Not only did the incidence of combined opioid-stimulant OHCA increase, but this specific combination was particularly lethal and was associated with a lower odds of survival compared with other drug profiles.

We observed a temporal increase in the incidence of OD-OHCA, particularly in the combination of opioid- and stimulant-related OHCA. These findings comport with other reports of OD epidemiology identifying an overall increase in substance use–related mortality,^18,19,20^ as well as a shift in OD profiles with increasing prevalence of synthetic opioids and stimulants.^21,22,23^ The increase in stimulant use with concomitant opioid use may be attributed to the ease of access to stimulants, the potential for the combination to enhance euphoria or reduce the untoward depressive effects of opioid, or the surreptitious adulteration of stimulants with synthetic opioids, such as fentanyl.^24,25^ While there has been deserved focus on public health measures and resuscitation guidelines for opioid-related OD,^2^ our study underscores the need for investment in public health infrastructure and prevention for these alternative drug profiles.^26,27^

In this study, the OD-OHCA group was younger, less likely to be witnessed, and less likely to have a shockable rhythm compared with non-OD OHCA group, characteristics consistent with prior research of aggregated OD-OHCA.^7,28^ Bystander CPR was common in the OD-OHCA group and comparable to the non-OD OHCA group in the study. These data are distinguished from previous studies in which bystander resuscitation for OD was uncommon and less frequent compared with non-OD OHCA.^7,28^ Greater bystander action in this study community may be due, in part, to the telecommunicator efforts to identify cardiac arrest and coach early CPR, which is a strong programmatic component of the study system’s strategy for resuscitation.^29^ The high prevalence of bystander action and the quick EMS response in this OD-OHCA cohort helps frame the observed survival outcomes, which may reflect the upper bound for this vulnerable OHCA group when using existing resuscitation strategies.

Importantly, the study results indicate that OD-OHCA is not a singular entity but instead can manifest important differences in presentation and outcome according to specific drug profiles. When stratified by the drug-specific profile, stimulant-only OHCA was more likely to be witnessed, to occur in a public location, and to present with a shockable rhythm. This presentation profile may be a consequence of the stimulant’s behavioral effects—activating the patient such that their arrest is witnessed—as well as the proarrhythmic effects of adrenergic excess related to stimulant use. However, these favorable characteristics associated with witnessed status and shockable initial rhythm did not translate to better survival outcomes for patients with stimulant-only OHCA compared with patients with OHCA with other drug profiles, highlighting the enhanced lethality of stimulants that might challenge resuscitation. While we did not have access to information related to long-term substance use prior to OHCA, previous studies have found an association between long-term stimulant use and cardiovascular pathology, including atherosclerosis and cardiomyopathy.^30^ The extent to which this underlying pathology further challenges resuscitation and outcomes in the setting of stimulant-only OHCA is unknown but may have implications for the timing and scope of preventive measures in this population.^31,32^

We observed poorer outcomes among patients with OHCA with combined opioid and stimulant use. Despite similar EMS response and treatment profiles to other OD groups, this combined group was less likely to be alive at the end of EMS care and had lower survival to hospital discharge. Even when considering patients with OHCA who survived to hospital admission, only one-third of those with combined stimulant-opioid OHCA survived to hospital discharge compared with the approximately 50% of patients with other OD OHCA profiles and non-OD OHCA who survived. This finding of worse survival after OHCA is in keeping with the general secular trend of increased all-cause mortality associated with combined opioid and stimulant use.^25,33,34,35^ Although the precise basis for increased OHCA mortality in this combined OD profile is not certain, the cumulative consequences of untoward respiratory and cardiac effects may combine to challenge heart resuscitation and brain recovery and ultimately increase case fatality. For those with long-term stimulant use, it is also possible that an underlying cardiac pathology may further reduce cardiac reserve and challenge resuscitation efforts following OHCA in the setting of combined opioid and stimulant overdose.^32^

### Limitations

There are limitations to the study. First, the investigation is an observational retrospective cohort study from a single, large metropolitan EMS system. Hence, the observed characteristics of the patients with OHCA and their resuscitation characteristics reflect the provincial OD epidemiology and EMS infrastructure in King County, Washington, and may not generalize more broadly, although the public health challenge associated with OD is common and EMS response for OD has increased throughout the US. Second, race and ethnicity were not ascertained in this cohort. Therefore, how these findings differ according to race and ethnicity or how they may generalize to populations with a different racial and ethnic distribution are not known. Third, although the study partnered with the MEO to undertake a systematic and rigorous approach to drug identification, some misclassification may have occurred. For example, drug-specific information was obtained through hospital-based urine toxicology and MEO-investigated postmortem blood samples, such that the data source differed for cases depending on survival status. Blood and urine toxicology can vary substantially due to differences in metabolic and kidney clearance.^36^ The MEO typically performs a toxicology evaluation for patients who are hospitalized if there are admission blood samples. Of note, we observed good agreement when information was available from hospital-based sources and MEO-based toxicology. To the extent that urine toxicology was not performed for all survivors of OHCA, there is a possibility that some cases of OD-OHCA would have been classified as non-OD OHCA. A similar direction of misclassification could have also occurred because some synthetic drugs may not be identified using contemporary toxicology screens. Conversely, we acknowledge that the presence of OD drug may be associated with clinically indicated use (eg, opioid use for chronic pain disorder) and may not be etiological in OHCA. While systematic attempts were made to adjudicate OD-OHCA at the exclusion of other plausible mechanisms, it is possible that some instances of OD-OHCA may have been misclassified. Finally, the multivariable analyses comparing OD-OHCA and non-OD OHCA were not primarily designed to advance the inference that survival is better or worse according to OD vs non-OD status, but rather they demonstrate the substantial measured differences in patient and presentation characteristics of OD-OHCA that can confound outcome relationships.

## Conclusions

In this population-based cohort study of patients with OHCA, we observed a significant increase in the incidence of OD-OHCA from 2015 to 2021, particularly among patients with OD-OHCA involving combined opioid and stimulant use. The similar unadjusted survival of patients with non-OD OHCA and patients with OD-OHCA belies differences in patient characteristics and resuscitation profiles, highlighting specific challenges to OD resuscitation. Moreover, presentation and outcome differ according to drug-specific etiology. In particular, combined stimulant-opioid OHCA was associated with lower odds of survival in unadjusted and adjusted analyses, a troubling finding given the increasing incidence of this drug-specific OHCA. Urgent public health resources and initiatives are needed to better understand, provide treatment for, and prevent combined stimulant-opioid OHCA.

## Notes

Supplement 1. **eMethods.** Comprehensive List of Compounds Identified in Toxicology and Distribution in Drug Profiles With Opioids **eTable 1.** Source of Out of Hospital Cardiac Arrest Decedent Toxicology Information–Medical Examiner Office Versus Healthcare Provider–Stratified by Age **eTable 2.** Crude Counts of Cardiac Arrest by Overdose Category and Age by Year **eTable 3.** Incidence Rate of Out of Hospital Cardiac Arrest in King County Between 2015 and 2021 Stratified by Overdose Drug Status **eTable 4.** Crude Counts of Cardiac Arrest by Drug-Specific Profile Category and Age by Year **eTable 5.** Incidence Rate of Out of Hospital Cardiac Arrest in King County Between 2015 and 2021 Stratified by Overdose Drug-Specific Profiles

Supplement 2.
Data Sharing Statement
