# Supplementary material for: Presentation and Outcomes of Adults With Overdose-Related Out-of-Hospital Cardiac Arrest
Source: JAMA Netw Open. 2023 Nov 7;6(11):e2341921. doi: 10.1001/jamanetworkopen.2023.41921 (PMC10630895; doi:10.1001/jamanetworkopen.2023.41921)
Supplement: Supplement 1. — eMethods. Comprehensive List of Compounds Identified in Toxicology and Distribution in Drug Profiles With Opioids eTable 1. Source of Out of Hospital Cardiac Arrest Decedent Toxicology Information–Medical Examiner Office Versus Healthcare Provider–Stratified by Age eTable 2. Crude Counts of Cardiac Arrest by Overdose Category and Age by Year eTable 3. Incidence Rate of Out of Hospital Cardiac Arrest in King County Between 2015 and 2021 Stratified by Overdose Drug Status eTable 4. Crude Counts of Cardiac Arrest by Drug-Specific Profile Category and Age by Year eTable 5. Incidence Rate of Out of Hospital Cardiac Arrest in King County Between 2015 and 2021 Stratified by Overdose Drug-Specific Profiles [file jamanetwopen-e2341921-s001.pdf]

Supplemental Online Content

Yogeswaran V, Drucker C, Kume K, et al. Presentation and outcomes of adults with overdose-related out-of-hospital cardiac arrest. *JAMA Netw Open*. 2023;6(11):e2341921. doi:10.1001/jamanetworkopen.2023.41921

eMethods. Comprehensive List of Compounds Identified in Toxicology and Distribution in Drug Profiles With Opioids

eTable 1. Source of Out of Hospital Cardiac Arrest Decedent Toxicology Information–Medical Examiner Office Versus Healthcare Provider–Stratified by Age

eTable 2. Crude Counts of Cardiac Arrest by Overdose Category and Age by Year

eTable 3. Incidence Rate of Out of Hospital Cardiac Arrest in King County Between 2015 and 2021 Stratified by Overdose Drug Status

eTable 4. Crude Counts of Cardiac Arrest by Drug-Specific Profile Category and Age by Year

eTable 5. Incidence Rate of Out of Hospital Cardiac Arrest in King County Between 2015 and 2021 Stratified by Overdose Drug-Specific Profiles

This supplemental material has been provided by the authors to give readers additional information about their work.

© 2023 Yogeswaran V et al. *JAMA Network Open.*

eMethods. Comprehensive List of Compounds Identified in Toxicology and Distribution in Drug Profiles With Opioids

1- Fentanyl and analogs
2- Heroin
3- Hydromorphone
4- Methadone
5- Morphine
6- Oxycodone
7- Opiates (other or unspecified)
8- Amphetamine
9- Methamphetamine
10- Cocaine (including crack cocaine)
11- Barbiturates
12- Benzodiazepines
13- Ecstasy/MDMA
14- Ketamine
15- Psilocybin
16- Marijuana
17- Spice
18- Synthetic Opioids
19- Dextromethorphan
20- Salvia Divinorum
21- Ethanol
22- Beta-Blocker
23- Diltiazem
24- Tylenol
25- Trazodone
26- Tricyclic Antidepressants
27- Seroquel/Quetiapine
28- PCP
29- Lithium
30- Valproate
31- SSRI
32- Flecainide
33- Tizanidine
34- Gabapentin
35- Lamotrigine
36- Venlafaxine
37- Isoniazid
38- Hydroxychloroquine
39- Hydroxyzine
40- Zolpidem
41- Hydrocodone
42- Diphenhydramine
43- 1,1 difluoroethane
44- Amlodipine

© 2023 Yogeswaran V et al. *JAMA Network Open*.

45- Mitragynine
46- Topiramate
47- Pregabalin
48- Pseudoephedrine
49- Cyclobenzaprine
50- Promethazine
51- Risperidone
52- Tramadol
53- Methocarmabol
54- Bupropion
55- Aripirazole
56- Ephedrine
57- Mirtapazine
58- Buprenorphine
59- Olanzapine
60- Cymbalta/Duloxetine
61- Xylazine
62- Isopropanol
63- Chlorpheniramine
64- MDA
65- Doxylamine
66- Tapentadol
67- Meprobamate
68- Meperidine
69- Doxepin

Of 295 cases of opioid OHCA, the distribution was 112 fentanyl, 77 heroin, 6 hydromorphone, 35 methadone, 10 morphine, 60 oxycodone, 30 other opiates, 5 hydrocodone and 1 synthetic opioid. In 205 cases of combined opioid + stimulant OHCA, the salient opioid was 75 fentanyl, 110 heroin, 3 hydromorphone, 24 methadone, 3 morphine, 13 oxycodone, 18 other opiates, and 1 hydrocodone.

© 2023 Yogeswaran V et al. *JAMA Network Open*.

eTable 1. Source of Out of Hospital Cardiac Arrest Decedent Toxicology Information–Medical Examiner Office Versus Healthcare Provider–Stratified by Age

	**OD-OHCA**				**Non-OD OHCA**			
	MEO jurisdiction		Healthcare Provider		MEO jurisdiction		Healthcare Provider	
Age Category	*N*	%	*n*	%	*n*	%	*n*	%
18-24	65	100	0	0	43	83	9	17
25-44	256	98	4	2	248	65	134	35
45-64	205	97	6	3	517	32	1115	68
65 or greater	26	96	1	4	356	12	2567	88
Total Population	552	98	11	2	1164	23	3825	77

OD, overdose; OHCA, out of hospital cardiac arrest; MEO, medical examiner office.

© 2023 Yogeswaran V et al. *JAMA Network Open*.

eTable 2. Crude Counts of Cardiac Arrest by Overdose Category and Age by Year

Subgroup	Year	Age Category, years	Count of Out of Hospital Cardiac Arrest	Population
OD OHCA	2015	18-24	8	104745
OD OHCA	2015	25-44	24	397529
OD OHCA	2015	45-64	22	391425
OD OHCA	2015	65 or greater	1	183482
OD OHCA	2016	18-24	7	109574
OD OHCA	2016	25-44	41	422536
OD OHCA	2016	45-64	29	387656
OD OHCA	2016	65 or greater	3	183853
OD OHCA	2017	18-24	8	110492
OD OHCA	2017	25-44	36	437028
OD OHCA	2017	45-64	38	387768
OD OHCA	2017	65 or greater	4	188813
OD OHCA	2018	18-24	17	110266
OD OHCA	2018	25-44	37	449578
OD OHCA	2018	45-64	43	388592
OD OHCA	2018	65 or greater	9	195620
OD OHCA	2019	18-24	11	109837
OD OHCA	2019	25-44	49	462372
OD OHCA	2019	45-64	35	389095
OD OHCA	2019	65 or greater	5	202260
OD OHCA	2020	18-24	15	109949
OD OHCA	2020	25-44	58	476657
OD OHCA	2020	45-64	44	391872
OD OHCA	2020	65 or greater	7	210388
OD OHCA	2021	18-24	22	110605
OD OHCA	2021	25-44	69	478305
OD OHCA	2021	45-64	54	391248
OD OHCA	2021	65 or greater	6	215385
Non-OD OHCA	2015	18-24	9	104745
Non-OD OHCA	2015	25-44	67	397529
Non-OD OHCA	2015	45-64	252	391425
Non-OD OHCA	2015	65 or greater	452	183482
Non-OD OHCA	2016	18-24	8	109574
Non-OD OHCA	2016	25-44	56	422536
Non-OD OHCA	2016	45-64	312	387656
Non-OD OHCA	2016	65 or greater	504	183853
Non-OD OHCA	2017	18-24	6	110492

© 2023 Yogeswaran V et al. *JAMA Network Open*.

Non-OD OHCA	2017	25-44	71	437028
Non-OD OHCA	2017	45-64	291	387768
Non-OD OHCA	2017	65 or greater	425	188813
Non-OD OHCA	2018	18-24	13	110266
Non-OD OHCA	2018	25-44	62	449578
Non-OD OHCA	2018	45-64	306	388592
Non-OD OHCA	2018	65 or greater	487	195620
Non-OD OHCA	2019	18-24	14	109837
Non-OD OHCA	2019	25-44	77	462372
Non-OD OHCA	2019	45-64	300	389095
Non-OD OHCA	2019	65 or greater	492	202260
Non-OD OHCA	2020	18-24	10	109949
Non-OD OHCA	2020	25-44	80	476657
Non-OD OHCA	2020	45-64	318	391872
Non-OD OHCA	2020	65 or greater	501	210388
Non-OD OHCA	2021	18-24	13	110605
Non-OD OHCA	2021	25-44	109	478305
Non-OD OHCA	2021	45-64	341	391248
Non-OD OHCA	2021	65 or greater	512	215385

OD, overdose; OHCA, out of hospital cardiac arrest

© 2023 Yogeswaran V et al. *JAMA Network Open*.

eTable 3. Incidence Rate of Out of Hospital Cardiac Arrest in King County Between 2015 and 2021 Stratified by Overdose Drug Status

**Total Population (N)**	1620158	1663224	1703773	1737314	1769326	1799945	1799945	
	**Age-Adjusted Incidence Rate per 100,000 (95% Confidence Interval)**							**P, linear trend**
**Non-OD OHCA**	69.1(64.2-73.9)	77.0(71.9-82.1)	68.0(63.3-72.7)	73.0(68.1-77.8)	72.9 (68.0-77.6)	72.7(68.0-77.6)	77.2(77.4-82.1)	0.30
**OD OHCA**	5.2(3.8-6.6)	7.2 (5.6-8.8)	7.5(5.9-9.1)	9.4(7.6-11.2)	8.76.9-10.4)	10.6(8.7-12.5)	13.0(10.8-15.0)	<0.01

© 2023 Yogeswaran V et al. *JAMA Network Open*.

eTable 4. Crude Counts of Cardiac Arrest by Drug-Specific Profile Category and Age by Year

Subgroup	Year	Age Category, years	Count of Out of Hospital Cardiac Arrests	Population
Opioid	2015	18-24	5	104745
Opioid	2015	25-44	10	397529
Opioid	2015	45-64	11	391425
Opioid	2015	65 or greater	1	183482
Opioid	2016	18-24	2	109574
Opioid	2016	25-44	21	422536
Opioid	2016	45-64	14	387656
Opioid	2016	65 or greater	1	183853
Opioid	2017	18-24	4	110492
Opioid	2017	25-44	17	437028
Opioid	2017	45-64	12	387768
Opioid	2017	65 or greater	1	188813
Opioid	2018	18-24	6	110266
Opioid	2018	25-44	11	449578
Opioid	2018	45-64	17	388592
Opioid	2018	65 or greater	3	195620
Opioid	2019	18-24	8	109837
Opioid	2019	25-44	23	462372
Opioid	2019	45-64	10	389095
Opioid	2019	65 or greater	3	202260
Opioid	2020	18-24	8	109949
Opioid	2020	25-44	24	476657
Opioid	2020	45-64	14	391872
Opioid	2020	65 or greater	4	210388
Opioid	2021	18-24	11	110605
Opioid	2021	25-44	32	478305
Opioid	2021	45-64	17	391248
Opioid	2021	65 or greater	5	215385
Stimulant	2015	18-24	1	104745
Stimulant	2015	25-44	5	397529
Stimulant	2015	45-64	3	391425
Stimulant	2015	65 or greater	0	183482
Stimulant	2016	18-24	1	109574
Stimulant	2016	25-44	4	422536
Stimulant	2016	45-64	9	387656
Stimulant	2016	65 or greater	1	183853
Stimulant	2017	18-24	2	110492

© 2023 Yogeswaran V et al. *JAMA Network Open*.

Stimulant	2017	25-44	4	437028
Stimulant	2017	45-64	8	387768
Stimulant	2017	65 or greater	1	188813
Stimulant	2018	18-24	1	110266
Stimulant	2018	25-44	5	449578
Stimulant	2018	45-64	12	388592
Stimulant	2018	65 or greater	0	195620
Stimulant	2019	18-24	0	109837
Stimulant	2019	25-44	6	462372
Stimulant	2019	45-64	9	389095
Stimulant	2019	65 or greater	0	202260
Stimulant	2020	18-24	3	109949
Stimulant	2020	25-44	12	476657
Stimulant	2020	45-64	14	391872
Stimulant	2020	65 or greater	1	210388
Stimulant	2021	18-24	1	110605
Stimulant	2021	25-44	10	478305
Stimulant	2021	45-64	16	391248
Stimulant	2021	65 or greater	0	215385
Stimulant + Opioid	2015	18-24	2	104745
Stimulant + Opioid	2015	25-44	7	397529
Stimulant + Opioid	2015	45-64	4	391425
Stimulant + Opioid	2015	65 or greater	0	183482
Stimulant + Opioid	2016	18-24	4	109574
Stimulant + Opioid	2016	25-44	9	422536
Stimulant + Opioid	2016	45-64	3	387656
Stimulant + Opioid	2016	65 or greater	0	183853
Stimulant + Opioid	2017	18-24	2	110492
Stimulant + Opioid	2017	25-44	12	437028
Stimulant + Opioid	2017	45-64	8	387768
Stimulant + Opioid	2017	65 or greater	0	188813
Stimulant + Opioid	2018	18-24	6	110266
Stimulant + Opioid	2018	25-44	19	449578
Stimulant + Opioid	2018	45-64	7	388592
Stimulant + Opioid	2018	65 or greater	3	195620
Stimulant + Opioid	2019	18-24	3	109837
Stimulant + Opioid	2019	25-44	18	462372
Stimulant + Opioid	2019	45-64	12	389095
Stimulant + Opioid	2019	65 or greater	2	202260
Stimulant + Opioid	2020	18-24	3	109949
Stimulant + Opioid	2020	25-44	18	476657

© 2023 Yogeswaran V et al. *JAMA Network Open*.

Stimulant + Opioid	2020	45-64	10	391872
Stimulant + Opioid	2020	65 or greater	1	210388
Stimulant + Opioid	2021	18-24	7	110605
Stimulant + Opioid	2021	25-44	23	478305
Stimulant + Opioid	2021	45-64	21	391248
Stimulant + Opioid	2021	65 or greater	1	215385
Other Drug	2015	18-24	0	104745
Other Drug	2015	25-44	2	397529
Other Drug	2015	45-64	4	391425
Other Drug	2015	65 or greater	0	183482
Other Drug	2016	18-24	0	109574
Other Drug	2016	25-44	7	422536
Other Drug	2016	45-64	3	387656
Other Drug	2016	65 or greater	1	183853
Other Drug	2017	18-24	0	110492
Other Drug	2017	25-44	3	437028
Other Drug	2017	45-64	10	387768
Other Drug	2017	65 or greater	2	188813
Other Drug	2018	18-24	4	110266
Other Drug	2018	25-44	2	449578
Other Drug	2018	45-64	7	388592
Other Drug	2018	65 or greater	3	195620
Other Drug	2019	18-24	0	109837
Other Drug	2019	25-44	2	462372
Other Drug	2019	45-64	4	389095
Other Drug	2019	65 or greater	0	202260
Other Drug	2020	18-24	1	109949
Other Drug	2020	25-44	4	476657
Other Drug	2020	45-64	6	391872
Other Drug	2020	65 or greater	1	210388
Other Drug	2021	18-24	3	110605
Other Drug	2021	25-44	4	478305
Other Drug	2021	45-64	0	391248
Other Drug	2021	65 or greater	0	215385

© 2023 Yogeswaran V et al. *JAMA Network Open*.

eTable 5. Incidence Rate of Out of Hospital Cardiac Arrest in King County Between 2015 and 2021 Stratified by Overdose Drug-Specific Profiles

**Total Population (N)**	1620158	1663224	1703773	1737314	1769326	1799945	1799945	
	**Age-Adjusted Incidence Rate per 100,000 (95% confidence interval)**							**P, linear trend**
**OD OHCA**	5.2(3.8-6.6)	7.2 (5.6-8.8)	7.5(5.9-9.1)	9.4(7.6-11.2)	8.76.9-10.4)	10.6(8.7-12.5)	13.0(10.8-15.0)	<0.01
**Opioid only**	2.6(1.6-3.5)	3.4(2.3-4.5)	3.0(2.0-4.1)	3.3(2.2-4.3)	4.0 (2.8-5.1)	4.4(3.1-5.6)	5.7(4.3-7.1)	<0.01
**Opioid and Stimulant**	1.3 (0.6-2.0)	1.6 (0.8-2.3)	2.0(1.1-2.8)	2.0(1.3-2.7)	3.2(2.1-4.3)	2.7(1.8-3.7)	4.4 (3.2-5.7)	<0.01
**Stimulant only**	0.9(0.3-1.4)	1.3 (0.6-1.9)	1.3 (0.6-2.0)	1.5 (0.8-2.2)	1.2 (0.6-1.8)	2.5 (1.6-3.4)	2.2 (1.4-3.0)	0.01
**Non-opioid, non-stimulant**	0.5(0.1-0.9)	1.0(0.4-1.6)	1.2 (0.6-1.9)	1.4 (0.7-2.2)	0.5 (0.1-0.9)	1.0(0.4-1.6)	0.7(0.2-1.2)	0.88

© 2023 Yogeswaran V et al. *JAMA Network Open*.
