# Supplementary material for: Presentation and Outcomes of Adults With Overdose-Related Out-of-Hospital Cardiac Arrest
Source: JAMA Netw Open. 2023 Nov 7;6(11):e2341921. doi: 10.1001/jamanetworkopen.2023.41921 (PMC10630895; doi:10.1001/jamanetworkopen.2023.41921)
Supplement: Supplement 2. — Data Sharing Statement [file jamanetwopen-e2341921-s002.pdf]

Data Sharing Statement

Yogeswaran. Presentation and Outcomes of Adults With Overdose-Related Out-of-Hospital Cardiac Arrest. *JAMA Netw Open*. Published November 07, 2023.
doi:10.1001/jamanetworkopen.2023.41921

Data
Data available: Yes
Data types: Deidentified participant data
How to access data: Data Sharing Statement Data Data available: Yes Data types: Deidentified participant data How to access data: Patient-level data will be available for reasonable requests through contacting the corresponding author When available: With publication Supporting Documents Document types: None Additional Information Who can access the data: Researchers whose proposed use of the data has been approved Types of analyses: Any purpose Mechanisms of data availability: After approval of a proposal and a signed data access agreement
When available: With publication

Supporting Documents
Document types: None

Additional Information
Who can access the data: Data Sharing Statement Data Data available: Yes Data types: Deidentified participant data How to access data: Patient-level data will be available for reasonable requests through contacting the corresponding author When available: With publication Supporting Documents Document types: None Additional Information Who can access the data: Researchers whose proposed use of the data has been approved Types of analyses: Any purpose Mechanisms of data availability: After approval of a proposal and a signed data access agreement
Types of analyses: Data Sharing Statement Data Data available: Yes Data types: Deidentified participant data How to access data: Patient-level data will be available for reasonable requests through contacting the corresponding author When available: With publication Supporting Documents Document types: None Additional Information Who can access the data: Researchers whose proposed use of the data has been approved Types of analyses: Any purpose Mechanisms of data availability: After approval of a proposal and a signed data access agreement
Mechanisms of data availability: Data Sharing Statement Data Data available: Yes Data types: Deidentified participant data How to access data: Patient-level data will be available for reasonable requests through contacting the corresponding author When available: With publication Supporting Documents Document types: None Additional Information Who can access the data: Researchers whose proposed use of the data has been approved Types of analyses: Any purpose Mechanisms of data availability: After approval of a proposal and a signed data access agreement
